# New real-time bowel sound analysis may predict disease severity in septic patients

**DOI:** 10.1186/cc14082

**Published:** 2015-03-16

**Authors:** J Goto, K Matsuda, N Harii, T Moriguchi, M Yanagisawa, D Harada, H Sugawara, O Sakata

**Affiliations:** 1 University of Yamanashi School of Medicine, Chuo, Japan; 2 University of Yamanashi, Kofe, Japan

## Introduction

Healthy bowel function is an important factor when judging the advisability of early enteral nutrition in critically ill patients, but long-term observation and objective evaluation of gastrointestinal motility are difficult. We developed a non-invasive monitoring system capable of quantifying and visualizing gastrointestinal motility in real time. In the study gastrointestinal motility was performed in patients with severe sepsis using this developed bowel sound analysis system, and the correlation between bowel sounds and changes over time in blood concentrations of IL-6, which is associated with sepsis severity, was evaluated.

## Methods

The study was a prospective, observational pilot study conducted in our hospital. Consecutive adult patients with severe sepsis, on a mechanical ventilator with an IL-6 blood concentration ≥100 pg/ml in the acute phase, defined as being up to the 28th day of illness in the ICU, were entered in this study between June 2011 and December 2012. Subjects were divided into those who were treated with steroids (steroid treatment group) and those who were not (no-steroids group) during the target period, because steroids strongly affect IL-6 blood levels.

## Results

The subjects were five adult patients in the acute phase of severe sepsis on a mechanical ventilator. Gastrointestinal motility was measured for a total of 62,399 minutes: 31,544 minutes in three subjects in the no-steroids group and 30,855 minutes in two subjects in the steroid treatment group. In the no-steroids group, the bowel sound counts were negatively correlated with IL-6 blood concentration (*r *= -0.76, *P *< 0.01), suggesting that gastrointestinal motility was suppressed as IL-6 blood concentration increased. However, in the steroid treatment group, gastrointestinal motility showed no correlation with IL-6 blood concentration (*r *= -0.25, *P *= 0.27). The IL-6 blood concentration appears to have decreased with steroid treatment irrespective of changes in the state of sepsis, whereas bowel sound counts with the monitoring system reflected the changes in the state of sepsis, resulting in no correlation.

## Conclusion

The new real-time bowel sound analysis system provides a useful method of continuously, quantitatively, and non-invasively evaluating gastrointestinal motility in severe patients. Furthermore, this analysis may predict disease severity in septic patients.

